# A multiplexed immunochemical microarray for the determination of cardiovascular disease biomarkers

**DOI:** 10.1007/s00604-023-06119-w

**Published:** 2023-12-27

**Authors:** Gloria Colom, Alejandro Hernandez-Albors, Jaume Barallat, Amparo Galan, Antoni Bayes-Genis, Juan-Pablo Salvador, Maria-Pilar Marco

**Affiliations:** 1 Nanobiotechnology for Diagnostics (Nb4D), Department of Chemical and Biomolecular Nanotechnology, Institute for Advanced Chemistry of Catalonia (IQAC) of the Spanish Council for Scientific Research (CSIC), Jordi Girona 18-26, 08034 Barcelona, Spain; 2 Centro de Investigación Biomédica en Red de Bioingeniería, Biomateriales y Nanomedicina (CIBER-BBN), Av. Monforte de Lemos, 3-5, 28029 Madrid, Spain; 3 Biochemistry Department, Metropolitan North Clinical Laboratory (LCMN), Germans Trias i Pujol Universitary Hospital, Ctra. de Canyet, s/n, Badalona, Barcelona, Spain; 4 Institut del Cor Germans Trias I Pujol, Ctra. de Canyet, 1-3, 08916 Badalona, Spain; 5 Centro de Investigación Biomédica en Red de Enfermedades Cardiovasculares (CIBERCV), Av. Monforte de Lemos, 3-5, 28029 Madrid, Spain

**Keywords:** Microarray, Fluorescence detection, Multiplexation, Heart failure, Acute myocardial infarction, Inflammation fluorescence, Immunoassay

## Abstract

A fluorescence antibody microarray has been developed for the determination of relevant cardiovascular disease biomarkers for the analysis of human plasma samples. Recording characteristic protein molecular fingerprints to assess individual’s states of health could allow diagnosis to go beyond the simple identification of the disease, providing information on its stage or prognosis. Precisely, cardiovascular diseases (CVDs) are complex disorders which involve different degenerative processes encompassing a collection of biomarkers related to disease progression or stage. The novel approach that we propose is a fluorescent microarray chip has been developed accomplishing simultaneous determination of the most significant cardiac biomarkers in plasma aiming to determine the CVD status stage of the patient. As proof of concept, we have chosen five relevant biomarkers, C-reactive protein (CRP) as biomarker of inflammation, cystatin C (CysC) as biomarker of renal failure that is directly related with heart failure, cardiac troponin I (cTnI) as already established biomarker for cardiac damage, heart fatty acid binding protein as biomarker of ischemia (H-FABP), and finally, NT-proBNP (N-terminal pro-brain natriuretic peptide), a well-established heart failure biomarker. After the optimization of the multiplexed microarray, the assay allowed the simultaneous determination of 5 biomarkers in a buffer solution reaching LODs of 15 ± 5, 3 ± 1, 24 ± 3, 25 ± 3, and 3 ± 1 ng mL^−1^, for CRP, CysC, H-FABP, cTnI, and NT-proBNP, respectively. After solving the matrix effect, and demonstrating the accuracy for each biomarker, the chip was able to determine 24 samples per microarray chip. Then, the microarray has been used on a small pilot clinical study with 29 plasma samples from clinical patients which suffered different CVD and other related disorders. Results show the superior capability of the chip to provide clinical information related to the disease in terms of turnaround time (1 h 30 min total assay and measurement) and amount of information delivered in respect to reference technologies used in hospital laboratories (clinical analyzers). Despite the failure to detect c-TnI at the reported threshold, the microarray technology could be a powerful approach to diagnose the cardiovascular disease at early stage, monitor its progress, and eventually providing information about an eminent potential risk of suffering a myocardial infarction. The microarray chip here reported could be the starting point for achieving powerful multiplexed diagnostic technologies for the diagnosis of CVDs or any other pathology for which biomarkers have been identified at different stages of the disease.

**Graphical Abstract:**

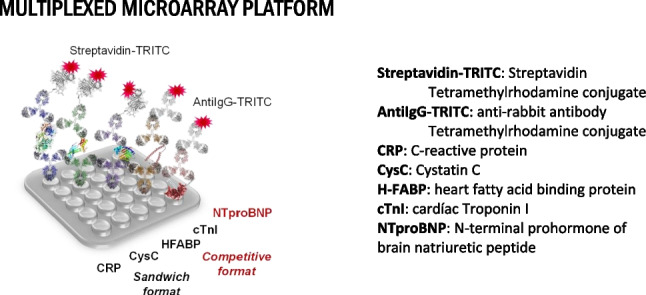

**Supplementary Information:**

The online version contains supplementary material available at 10.1007/s00604-023-06119-w.

## Introduction

Early, reliable, and accurate diagnostic tools of a specific disease are crucial for an effective treatment. This is particularly important at the point of care (PoC), where an immediate decision of the appropriate treatment needs to be made. Such is the case of for example an event of stroke, hair failure, or sepsis, where the rapid and precise confirmation of clinical findings is of vital relevance [1]. However, in many instances, clinical evidence based on a single biomarker is not adequate for an appropriate diagnosis of a disease or for monitoring its treatment. Often it is highly desirable to screen various analytes simultaneously, enabling a rapid, low-cost, and reliable quantification. Clinical diagnosis of many diseases depends on the accurate and unambiguous detection of various biomarkers, which may include proteins, peptides, small molecules, or nucleic acids. Therefore, multiplexing is becoming each time more important, particularly in the last decade [2].

According to World Health Organization (WHO) [3], cardiovascular diseases (CVDs) are a group of disorders of the heart and blood vessels which include coronary heart disease, acute myocardial infraction (AMI), heart failure (HF) or strokes, among others. Most of the CVD deaths are due to heart attacks and strokes, mainly a consequence of a blockage that prevents blood from flowing to the heart or brain. In the cardiovascular disease field, numerous studies searching for various biochemical markers have emerged in the last two decades [4–6]. A multiplexed technological approach detecting multiple markers would increase their diagnostic and prognostic value. Therefore, there is still a clear need for improvement in the direction to provide clinicians with test that provides a more complete information of the disease to assist them on decision-taken for those patients with chest discomfort and inconclusive electrocardiography.

**Fig. 1:**
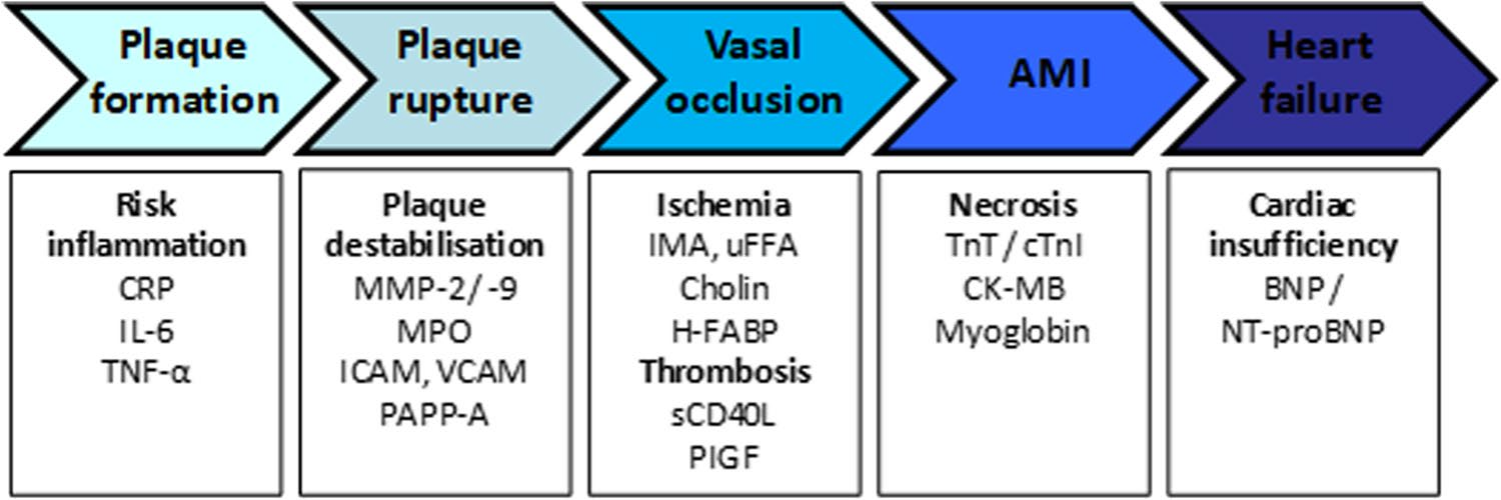
The entire pathophysiology of acute coronary syndrome (ACS). This flowchart depicts candidate markers related to earlier aspects of atherogenesis which may provide independent information in the diagnosis of AMI. CRP C-reactive protein, IL-6 interleukin-6, TNFα tumor necrosis factor α, MMP-2/-9 matrix metalloproteinases 2 and 9, MPO myeloperoxidase, ICAM intracellular cell adhesion molecules, VCAM vascular cell adhesion molecules, PAPP-A pregnancy-associated plasma protein A, IMA ischemia-modified albumin, uFFA unbound free fatty acids, H-FABP heart-type-isoform fatty acid binding protein, sCD40L soluble CD40 ligand, PlGF placental growth factor, TnT troponin T, cTnI cardiac troponin I, CK-MB creatine kinase MB isoenzyme, BNP brain natriuretic peptide, NT-proBNP N-terminal pro-brain natriuretic peptide. Figure adapted from [6]

Thus, different biomarkers are released to the blood stream depending on the stage of the cardiovascular event [7, 8] (see Fig. 1). Well-established biomarkers such as CRP (C-reactive protein), indicator of an inflammatory process, troponins, resulting from necrosis and brain natriuretic peptides related to cardiac insufficiency, have been recognized by the National Academy of Clinical Biochemistry (NACB) [9] and the European Society of Cardiology (ESC) [10] as reference biomarkers to evaluate the risk and diagnosis of different cardiovascular diseases (see Fig. 1). Other biomarkers such as interleukin-6 (IL-6), tumor necrosis factor α (TNF-α), myeloperoxidase (MPO), ischemia modified albumin (IMA), or soluble CD40 ligand (SD40L) have not implemented so far in the current protocols, however are indicative of processes related to the disease. Such is the case of the TNFα and IL-6 [11], inflammatory biomarkers or of the heart fatty acid binding protein (H-FABP) which has been recognized as a biomarker of ongoing myocardial damage [12].

Research in diagnostics seeks developing reliable multiplexed technologies able to map the health status of an individual based on the profile of a selected panel of biomarkers. On CVDs, this approach would allow a better stratification of the patients based on the information about the stage of the disease provided by biomarkers involved in the different stages from inflammation events to tissue injure or necrosis. Main challenges of multiplexed analysis [13] are derived from the variety of the chemical nature of the different biomarkers involved through the disease progress and also by the fact that each of them should be quantified at different threshold. An ideal multiplexed diagnostic tool should allow simultaneous quantification of the biomarkers in a single run. However, this is tricky considering that great broad range of concentrations that should be measured. For example, on CVDs, the blood serum levels of the inflammatory biomarker CRP may dramatically increase to up to 3000 ng mL^−1^ when there is high risk of heart injury. In contrast, cardiac troponin I (cTnI), considered the gold standard CVD biomarker, reaches levels in the 0.06 ng mL^−1^ identifying patients with ongoing cardiomyocyte necrosis who are at increased risk.

Immunoassays are standard diagnostic methods that have a key role in CVD. Hence, a great variety of diagnostic tools and devices do exists available nowadays for diagnosis of these CVDs at different stages [14], many of them based on the use of antibodies. Antibody-based analytical methods are characterized for their simplicity, speed, and generally for their sensitivity and specificity. There have been several attempts to develop immunochemical assays employing different technologies to accomplish the multi-detection of cardiovascular biomarkers, mainly panels of 2, maximum 3 or 4 biomarkers. In these panels, cTnI is mandatory, due to its specificity in respect to injury of the cardiac tissue. However, early prediction requires highly sensitive methods, which not always is possible, particularly on a multiplexed format. As an example, the lab-on-a-disk [15] consisted in novel centrifugal microfluidic layout that consists of three reaction chambers interconnected for the common processes such as sample injection, incubation, and washing and then isolated on-demand for the independent processes such as substrate incubation and final detection, and the screen-printed (SP) chemiluminescent microarray systems need to improve the detectability for cTnI, brain natriuretic peptide (BNP), and N-terminal pro-brain natriuretic peptide (NT-proBNP). Another example is an electrochemical biosensor based on a SP platform composed of eight working electrodes modified with electro-addressed protein A-aryl diazonium adducts [16]. The electrode surfaces are then used as an affinity immobilization support.

More recently, Guo et al. reported also a multiplexed platform using ZnO nanowires to enhance the fluorescence signal [17], which did not reach either the desired detectability for c-TnI. Conversely, the MSD MULTI-ARRAY™ (commercially available electrochemiluminescent microarray) incorporates a highly sensitive cTnI assay, as well as other authors that could achieved the required detectability involving useful strategies. Some of these strategies are the amplification of the magnetic signal once developing a magnetic immunochromatography [18], the involvement of a cationic isotachophoresis (ITP) to separate and concentrate 2 proteins with similar isoelectric points in a paper-based analytical device [19], performing a DNA microarray [20], or developing a nonfaradaic electrochemical assay [21].

Microarray technology holds great potential for multiplexation allowing manufacturing high density microarrays with a great capability to detect and quantify a wide collection of biomarkers through site-encoded codification on very small chip surfaces and with small sample volumes [22, 23]. Then, in this work, we report the investigations performed to the simultaneously determination of a selected panel of CVD biomarkers in plasma based on the combination of antibodies and immunoreagents on a fluorescent microarray chip. The development of a tool for the determination of the fingerprint of the CVD event is optimized and provided. A microarray has been developed in a 96-well plate format (24 samples per microarray chip), allowing the quantification of five biomarkers in the relevant disease range for each biomarker. The microarray has proven to be extraordinarily efficient in comparison to the benchtop analyzers commonly used in clinical laboratories. Optimization on the most suitable immunoreagents used as well as ensuring that the analytical parameters from individual to multiplexed assay remained.

## Materials and methods

### Chemicals and biochemicals

Most of the chemicals and biochemicals, otherwise indicated, are supplied from Sigma Chemical Co. (St. Louis, MO, USA). EZ-link sulfo-NHS-LC-LC-biotin was obtained from Pierce (Rockford, IL). Polyclonal goat antibodies for CRP and cystatin C (Cys C) and the corresponding standards were provided by Audit Diagnostics Ltd. (Cork, Ireland) and polyclonal sheep and monoclonal sheep anti-H-FABP from Randox Laboratories Ltd (Crumlin, UK). Polyclonal goat anti-cTnI, native cTnI, and human cardiac H-FABP were provided by Life Diagnostics Inc. (West Chester, PA). As220 was produce in the laboratory by the immunization of the native cTnI [24]. Human recombinant NT-proBNP and human cardiac troponin I-T-C complex were obtained from Hytest Ltd (Turku, Finland). The preparation of the labeled antibodies and competitors has been performed with the support of the U2 of the ICTS “NANBIOSIS,” more specifically by the Custom Antibody Service (CAbS, CIBER-BBN, IQAC-CSIC). All salts were provided by Merck (Darmstadt, Germany). The procedure for the preparation of NB1-SIA-BSA conjugate and for the production of As 251 is already described [25]. Goat Anti-Rabbit IgG H&L TRITC conjugate from Abcam Plc (Cambridge, UK). Streptavidin-TRITC was purchased from Thermo Scientific (Rockford, IL).

### Equipment

The pH and the conductivity of all buffers and solutions were measured with a pH meter pH 540 GLP and a conductimeter LF 340, respectively (WTW, Weilheim, Germany). Dilution plates were purchased from Nirco (Barberà del Vallés, Spain). Plain microscope glass slides (75 × 25 mm) were purchased from Corning Inc (Corning, NY, USA). The slide printing was done using a BioOdissey Calligrapher™ MiniArrayer (Bio-Rad Laboratories, Inc., Hercules, CA, USA). All microarrays assays were performed on an ArrayIt® holder (Arrayit Corp, Sunnyvale, CA, USA). Microarray measurements were recorded on a ScanArray Gx PLUS (PerkinElmer, Waltham, MA, USA) with a Cy3 optical filter with 10-µm resolution. The laser power and photomultiplier tube (PMT) gain were set to 95% and 80%, respectively. The excitation and emission wavelengths used were at 543 and 570 nm, respectively. The spots were measured by deducting the mean Cy3 background intensity to the mean of Cy3 foreground intensity using ScanArray Express v 4.0 (Microarray Analysis System, PerkinElmer, Waltham, MA, USA). For the microarray assay, four slides were placed on a 96-well microplate microarray hardware (ArrayIt®, Silicon Valley, CA, USA) provided with a silicon gasket defining 8 × 3 wells on each slide.

### Antibody biotinylation of AbCRP1-biotin, AbCysC-biotin, and AbH-FABP-biotin

A solution of EZ-link sulfo-NHS-LC-LC-biotin (0.34 mg) in Milli-Q water (200 μL) was added dropwise to an antibody solution (4 mg) in borate buffer (2 mL) already incubated in an ice bath at 4 °C. The mixture was stirred in the ice bath for 30 min and then at RT for 4 h. Final conjugates were purified by dialysis against PBS 0.5 mM (4 × 5 L) and Milli-Q water (1 × 5 L), lyophilized and stored at − 20 °C. Working aliquots (1 mg mL^−1^, PBS 10 mM) were kept at 4 °C.

### Microarray chips manufacturing

Plain glass slides were first cleaned by immersing them in piranha solution (H_2_SO_4_:H_2_O_2_, 70:30, v/v, 1 h), rinsed with ultrapure water, activated with 10% (w/v) NaOH (1 h), and then rinsed again with ultrapure water and absolute ethanol. Once the slides were cleaned, they were dried with N2, and the chemical functionalization of the slides was achieved immersing the glass slides in a solution of 2.5% (v/v) 3-glycidyloxypropyltrimethoxysilane (GPTMS) (3 h). Afterwards the slides were washed with ethanol, dried, and stored in the desiccator until use. Biofunctionalization was performed by spotting solutions of the antibodies or the bioconjugate competitors in printing buffer (0.5-1 nl spot-1) under controlled temperature (20 °C) and humidity (65%) using a solid pin. The slides were maintained for 1 h inside the microarrayer chamber. The biofunctionalized slides could be stored at RT in a desiccator until use. Up to 24 microarray chips could be printed in a single slide. Microarray chips for single analyte analysis had 5 spots, while for multiplexed analysis consisted of a matrix of 5 × 5 spots (5 analytes, 5 replicates).

### Microarray assay procedure

Both competitive and sandwich immunoassay calibration curves were analyzed with a four-parameter logistic equation using the software GraphPad Prism v 5 (GraphPad Software Inc., San Diego, CA, USA). The standard curves were fitted to a four-parameter equation according to the following formula: *Y* = [(*A* − *B*)/1 − (*x*/*C*)*D*] + *B*, where *A* is the maximal fluorescence, *B* the minimum fluorescence, *C* the concentration producing 50% of the difference between *A* and *B* (or IC50), and *D* the slope at the inflection point of the sigmoid curve. The limit of detection (LOD) for a competitive immunoassay was defined as the concentration producing 90% of the maximal fluorescence (IC90), while, for sandwich immunoassay, the IC10 (the concentration producing 10% of the maximal signal) value was considered to assess the detectability of the microarray. Unless otherwise indicated, the results of single-analyte microarray assays correspond to assay performed in 1 day using at least 5 spot replicates of each concentration whereas for the multiplexed microarray the results given were usually performed in 3 microarray chips performed on at least 3 different days and using five-spot replicates.

For the microarray assay, before starting the assay, the slides were washed four times with PBST. Standard solutions used to calibrate the microarray were prepared in 0.15% casein PBST or the corresponding dilution in plasma (7 concentration points plus zero: 1 to 4000 ng mL^−1^ for CRP, 0.1 to 500 ng mL^−1^ for CysC, 1 to 5000 ng mL^−1^ for H-FABP, 1 to 4000 ng mL^−1^ for Tn ICT complex, and 0.1 to 2000 ng mL^−1^ for NT-proBNP). Optimum concentrations of the bioconjugate competitors spotted and antisera/antibodies dilutions (see Table 1) were previously chosen by performing two-dimensional titration assays (2D assay) and selecting those conditions able to generate a signal above 10,000 RFUs (relative fluorescence units) at 70–80% of the saturation curve. For this purpose, the binding of serial dilutions of the antisera/antibodies (zero and 1/1000 to 1/64,000, using 100 μL well^−1^) to different concentrations of the bioconjugates spotted (zero and 200 μg mL^−1^ to 0.025 μg mL^−1^) was analyzed.

**Table 1:** Microarray analytical features for each analyte in both individual and multiplexed format assays. The calibration curves were performed in PBST 0.15% casein pH 7.5

	CRP^a^		Cys C^b^		H-FABP^c^		cTnITC^c^		NT-proBNP^c^	
	Abcapt: AbCRP2, 200 µg/mL Abdet: AbCRP1-biotin, 2 µg/mL		Abcapt: AbCysC, 200 µg/mL Abdet: AbCysC-biotin, 150 µg/mL		Abcapt: C3, 100 µg/mL Abdet: AbHFABP-biotin, 20 µg/mL		Abcapt: Goat anti-cTnI, 125 µg/mL Abdet: As220, 1/1000		Coating antigen: NB1-SIA-BSA: 100 µg/mL Abdet: As251, 1/400	
	Individual	Multiplexed^h^	Individual	Multiplexed^h^	Individual	Multiplexed^h^	Individual	Multiplexed^h^	Individual	Multiplexed^i^
RFU_max_ (·10^3^)	13.4 ± 2.7	12.4 ± 0.4	12.5 ± 2.1	8.9 ± 1.0	16.7 ± 1.4	7.1 ± 0.9	6.9 ± 0.3	11.1 ± 3.2	10.4 ± 1.4	9.2 ± 5.2
RFU_min_ (·10^3^)	0.5 ± 0.2	0.6 ± 0.2	0.5 ± 0.4	0.4 ± 0.1	0.1 ± 0.5	0.1 ± 0.2	0.2 ± 0.1	0.5 ± 0.2	0.9 ± 0.2217	0.6 ± 0.1
Slope	1.3 ± 0.2	1.5 ± 0.2	1.2 ± 0.2	1.3 ± 0.2	1.3 ± 0.2	1.57 ± 0.05	1.9 ± 0.3	1.9 ± 0.2	− 1.8 ± 0.3	− 1.3 ± 0.3
*R*^2^	0.94 ± 0.03	0.97 ± 0.01	0.96 ± 0.06	0.98 ± 0.01	0.96 ± 0.02	0.98 ± 0.01	0.92 ± 0.02	0.97 ± 0.02	0.94 ± 0.01	0.97 ± 0.02
IC50, ng mL^−1^	74 ± 15	72 ± 11	20 ± 3	19 ± 2	68 ± 5	100 ± 15	82 ± 13	83 ± 13	16 ± 4	24 ± 12
Working range, ng mL^−1^	233 ± 63/26 ± 5	181 ± 22/27 ± 7	62 ± 13/6.0 ± 0.9	50 ± 10 6 ± 1	220 ± 36/23 ± 4	252 ± 62/41 ± 6	191 ± 23/38 ± 5	202 ± 17/40 ± 4	7 ± 2/40 ± 6	6.9 ± 0.5/85 ± 62
LOD, ng mL^−1^	14 ± 3	15 ± 5	3.0 ± 0.6	3 ± 1	13 ± 3	24 ± 3	24 ± 4	25 ± 3	4.1 ± 0.8	3 ± 1
Cut-off, ng mL^−1^	1000–3000^e^ ˃3000^f^ ≥ 10,000^ g^		˃1290		˃5.8		˃0.005		˃0.3	

^a^Each curve was tested 6 times (interday and intraday) using at least 18 replicates on every analyte concentration (18 spots per well).^b^Each curve was tested 7 times (interday and intraday) using at least 18 replicates on every analyte concentration (18 spots per well). ^c^Each curve was tested 3 times (interday and intraday) using at least 18 replicates on every analyte concentration (18 spots per well). ^d^Each curve was tested twice (interday and intraday) using at least 12 replicates on every analyte concentration (12 spots per well). ^e^Average risk, ^f^high risk, ^g^very high risk. ^h^Each calibration curve was assayed three times in three different days using six replicates for each concentration point in every well (microarray). Each microarray was used to test a determined analyte concentration for all five targets. Thus, a calibration curve was obtained employing 8 different microarrays, each with different concentrations of the target analytes. The biofunctionalized slides used were from three different batches. ^i^All parameters were calculated from two replicates in two different days

The samples were split in two parts, one of them diluted 1/40 for CRP and Cys C, and the other measured undiluted for the rest of the biomarker targets. Solutions (100 µL/well) of the standards or the samples were added to the corresponding wells of the slide, incubated for 30 min at RT and washed four times with PBST. In this step, also As251 (1/400) was added as a detection antibody in the competitive assay for NT-proBNP determination. Then, solutions of single antibody or the Ab cocktail (in PBST, 100 µL/well) were added: Ab-CRP1-biotin (2 μg mL^−1^), AbCysC-biotin (20 μg mL^−1^), AbH-FABP-biotin (150 μg mL^−1^), and As220 (1/1000).

After 30 min of incubation at RT, the slides were washed 4 times with PBST, and a solution containing a mixture of streptavidin-TRITC and anti-rabbit IgG-TRITC conjugates (1/625 and 1/250 in PBST, respectively, 100 µL/well) was added. After another incubation of 30 min at RT in the dark, the slides were washed (3 × PBST + 1 × ultrapure water), dried with N_2_, and read with the microarray scanner as described above.

### Accuracy studies

The accuracy was assessed by measuring plasma samples fortified with all analytes at different concentrations. The samples were split in two parts, one of them diluted 1/40 for CRP and CysC, and the other measured undiluted for the rest of the biomarker targets. Analysis were performed in six different days for CRP and CysC, while for H-FABP, cTnITC complex, and NT-proBNP analyses were done in three different days employing at least 6 spot replicates per microarray for each target.

### Plasma samples

Clinical plasma samples obtained from patients showing different pathologies were provided by Institut d’Investigació Germans Trias i Pujol (IGTP) for the preliminary evaluation of the multiplexed microarray developed. These plasma samples had been already analyzed at the IGTP on benchtop analyzers where the different biomarkers were tested individually. cTnI and CRP concentrations had been analyzed using a Siemens Dimension analyzer, and for NT-proBNP with a Radiometer AQT 90 FLEX.

## Results and discussion

Microarrays are an excellent option for multiplexed immunoassays. Microarrays can be defined as a collection of microscopic spots spatially codified on a solid surface, in which bioreceptors have been immobilized (solid state or planar microarrays). Alternatively, multiplexed immunoassays can be carried out in solution by using other technological approaches such as codified beads (barcodes or non-planar microarrays) [26].

In this work, we have addressed the development of a fluorescent microarray chip for the simultaneous detection and quantification of a selected panel of CVD biomarkers (CRP, CysC, H-FABP, cTnI, and NT-proBNP). The biomarkers have been selected to provide a broad overview and a comprehensive information of the underlying pathology or the cardiovascular event the patient is suffering. From a well-established biomarker of inflammation like CRP [27], a biomarker of plaque rupture such as h-FABP [28], the golden standard cTnI for myocardial infarction to NT-proBNP for heart failure, adding CysC [29], a biomarker for renal failure, associated to have an increased cardiovascular risk profile. Thus, the biomarkers cover the pathology from early stages such as inflammation, to late stages provoking AMI and HF events [6]. Cut-off levels for CRP, CysC, H-FABP, cTnI, and NT-proBNP are 3000 ng mL^−1^, 1290 ng mL^−1^, 5.8 ng mL^−1^, 0.005 ng mL^−1^, and 0.3 ng mL^−1^, respectively.

The main challenges needed to be faced up with during the multiplexed assay development were that the biomarkers selected include a peptide (NT-proBNP) and four proteins (CRP, CysC, H-FABP, and cTnI) which are found in the blood stream at different concentration ranges in the case during the CVD progress. The different chemical properties of these biomarker targets could influence performance of the multiplexed assay. Hence, for example, the isoelectric point (pI) ranges from 6.40 for the case of CRP to 9.87 for cTnI (CysC, 9.30; h-FABP 6.34; and NT-proBNP 8.45), which may determine the assay conditions required to quantify each analyte. Moreover, the different size of the biomarkers requires different immunoassay formats, competitive format for NT-proBNP and sandwich format for CRP, CysC, H-FABP, and cTnI.

### Individual microarray assays

**Fig. 2:**
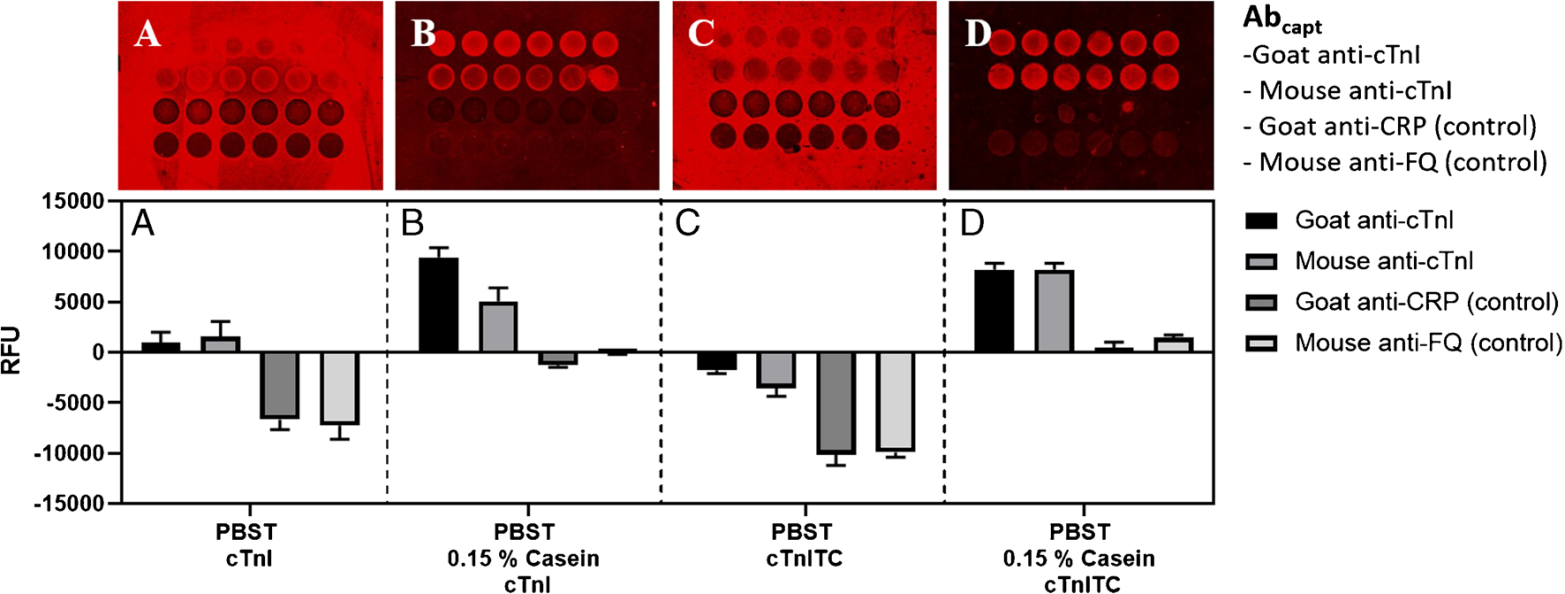
Evaluation of the cTnI and cTnI in I-T-C complex non-specific adsorption when 0.15% casein was present in the assay buffer (PBST). Both cTnI forms were assayed at 0.15 µg mL^−1^, using a [Abcapt] = 0.5 mg/mL and As220 as Abdet at 1/1000. The standard deviation shown is the result of analysis made 1 day using 6 well replicates

Key elements for setting up a multiplexed diagnostic kit are the availability of immunoreagents, appropriately labeled to provide a signal in the presence of the biomarker target, and the solid support in which the immunochemical reaction is going to take place. Recently, we have reported the development of antibodies and an ELISA for NT-proBNP biomarker using competitive immunoassay format [25]. Moreover, previously, we developed antibodies against cTnI and use them on a sandwich configuration to develop an electrochemical immunosensor [24]. Immunoreagents were also found available from different sources to set up immunochemical assays to detect the rest of the biomarker targets on a sandwich format (see Fig. 2). In order to standardize detection of the immunochemical reaction, the antibodies were biotinylated. Thus, the proper bioconjugates should be prepared as a competitor for NT-proBNP [25] and biotinylation of detection antibodies. The biotinylation was required in order to homogenize the detection antibodies (CRP, CysC, and h-FABP) that were from different immunized animals and avoid cross-reactivity for the capture antibodies. The biotinylated antibodies will be detected by the corresponding streptavidin-TRITC conjugate, while the detection antibodies for NT-proBNP and cTnI, we decided to use and anti-rabbit IgG-TRITC conjugate. After setting up the necessary concentrations of immunoreagents through checkerboard titration experiments, we were able to establish five individual microarray assays for the five target biomarkers selected.

The microarray assay protocol is described in the “Materials and methods” section. Some of the remarkable features of the assay is the preincubation step set in 15 min for all assays due to As251 (NT-proBNP antibody) required a preincubation between the specific antibody and the sample to improve the detectability assay [25]. In addition, 0.15% of casein was added to PBST buffer to reduce the background was observed in cTnI assay in microarray format. As it can be observed in Fig. 2A and C, and high background was observed in PBST that was minimized by using a blocking agent like 0.15% of casein in PBST that avoid that cTnI stick on the glass surface (Fig. 2B and D). However, the background was not completely avoided until it was used a ternary complex of cTnITC (cardiac troponin complexed with subunits I-T-C) which were not adsorbed to the glass slides. Thus, 0.15% casein in PBST was used in buffer assays (sample buffer) and used as a standard. cTnITC complex can be used as reference in troponin assay developments.

After the selection of the most suitable capture/detection antibodies as well BSA conjugate concentrations, a calibration curve for each biomarker was established. The features of those assays are shown in Table 1. According to the fitting parameters obtained, the LOD for CRP and CysC, 14 ± 3 and 3.0 ± 0.6 ng·mL^−1^, respectively, has enough detectability for their quantification. ECS and NACB stated a minimum concentration to be found in blood to be considered as a risk in 1000 and 1290 ng·mL^−1^ [30, 31]. So, a dilution of the sample is required to measure accurately. For H-FABP, the cut-off value to assess heart tissue damage is set on 5.8 ng/mL [32] which is in the range of the LOD found using the fluorescent microarray (13 ± 3 ng·mL^−1^). Concentration values in the range of 20.3–48.1 ng/mL for H-FABP predict more accurately the acute heart failure [33]. For NT-proBNP, the cut-off value is set in 0.3 ng/mL which is one order of magnitude lower than the assay developed on the microarray format (LOD 4.1 ± 0.8 ng mL^−1^), however NT-proBNP levels of patients suffering HF reach values of 2 ng/mL, therefore the microarray can be used to determine that the patient is on HF. cTnI assay cannot reach the required detectability for ESC and NACB in 0.05 ng·mL^−1^ [34]; however, in some cases, cTnI concentration after AMI is found 10–100 ng·mL^−1^ [35].

In comparison to the basal human blood levels of these biomarkers and the recommended cut-off points, it can be observed how, in buffer, the CRP and CysC microarrays were able to reach a detectability below the basal levels in healthy patients. However, it did not happen to H-FABP, cTnI, and NT-proBNP, being the cTnI assay the worst case. According the current guidelines and literature reporting the clinical levels in cardiac injury found in troponins [36, 37], H-FABP [38], and NT-proBNP [39] are higher than 10 ng/mL, up to 50 ng/mL, and higher than 2 ng/mL, respectively. Thus, we decided to face the analysis of the clinical samples aiming to define in which clinical stage is the patient within the CVD event. In that respect, a positive aspect was the lack of cross-reactivity towards the different biomarkers of each individual assay; all the assays were specific for its corresponding biomarker.

### Multiplexed cardiac microarray

**Fig. 3:**
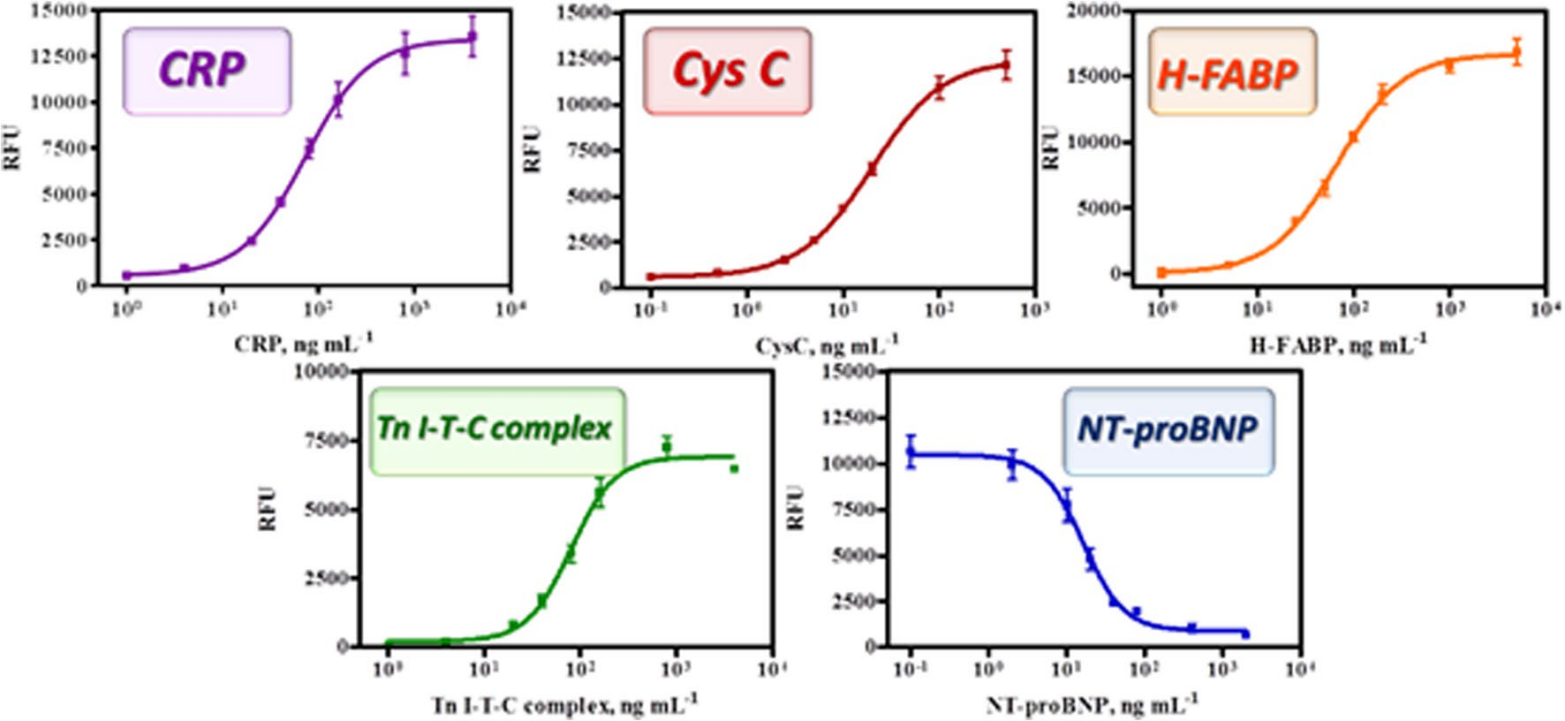
Calibration curves for each analyte: CRP, Cys C, H-FABP, cTnITC complex, and NT-proBNP in PBST 0.15% casein pH 7.5 in a multiplexed configuration

The conditions established for the individual microarrays were implemented in the multiplexed format. Performance of the multiplexed microarray was first assessed in buffer. The analytical parameters of the calibration curves obtained under these conditions are shown in Fig. 3 and summarized the analytical parameters in Table 1. Limit of detection reached are 15 ± 5, 3 ± 1, 24 ± 3, 25 ± 3, and 3 ± 1 ng mL^−1^ for CRP, CysC, H-FABP, cTnI, and NT-proBNP, respectively, in 1 h 30 min total assay and measurement. As it can be observed, the analytical parameters of the assays in the multiplexed format are very similar to those obtained when the individual assays. Only for H-FABP the values of the IC_50_ and of the LOD were slightly higher.

Due to the differences in the concentration ranges between the biomarkers selected, analysis had to be performed splitting the samples in two parts. Hence, for CRP and CysC, the samples had to be diluted in order to place them within the dynamic range of the assay. On the other side, the detectability of the assays for H-FABP, cTnITC, and NT-proBNP allowed direct analysis of the samples. Assessment of the performance of the multiplexed microarray in clinical samples was made using human plasma provided by Banc de Sang i Teixits de Catalunya. On a first instance, the potential non-specific interferences caused by these sample matrixes were evaluated (matrix effect studies). For this purpose, standard curves were prepared in buffer, plasma at the proper dilution factor. The expected concentration of CRP and CysC that can be found in the serum, it was very much likely that such effect was specific considering the detectability of our microarray and the basal levels which are considerably high: 1000 ng mL^−1^ for CRP (IC50 in buffer is around 70.5 ng mL^−1^) and between 800 and 1200 ng mL^−1^ for CysC approximately (IC50 in buffer is around 9.52 ng mL^−1^). Thus, we decided to dilute the sample 1/40 while for CRP and CysC determination and cTnI, H-FABP, and NT-proBNP in undiluted plasma. As it can be observed, the response of the microarray for H-FABP, Tn I-T-C complex, and NT-proBNP in undiluted plasma and CRP and CysC in 1/40 were not significantly affected by the matrix of interest (see Figure S1).

**Fig. 4:**
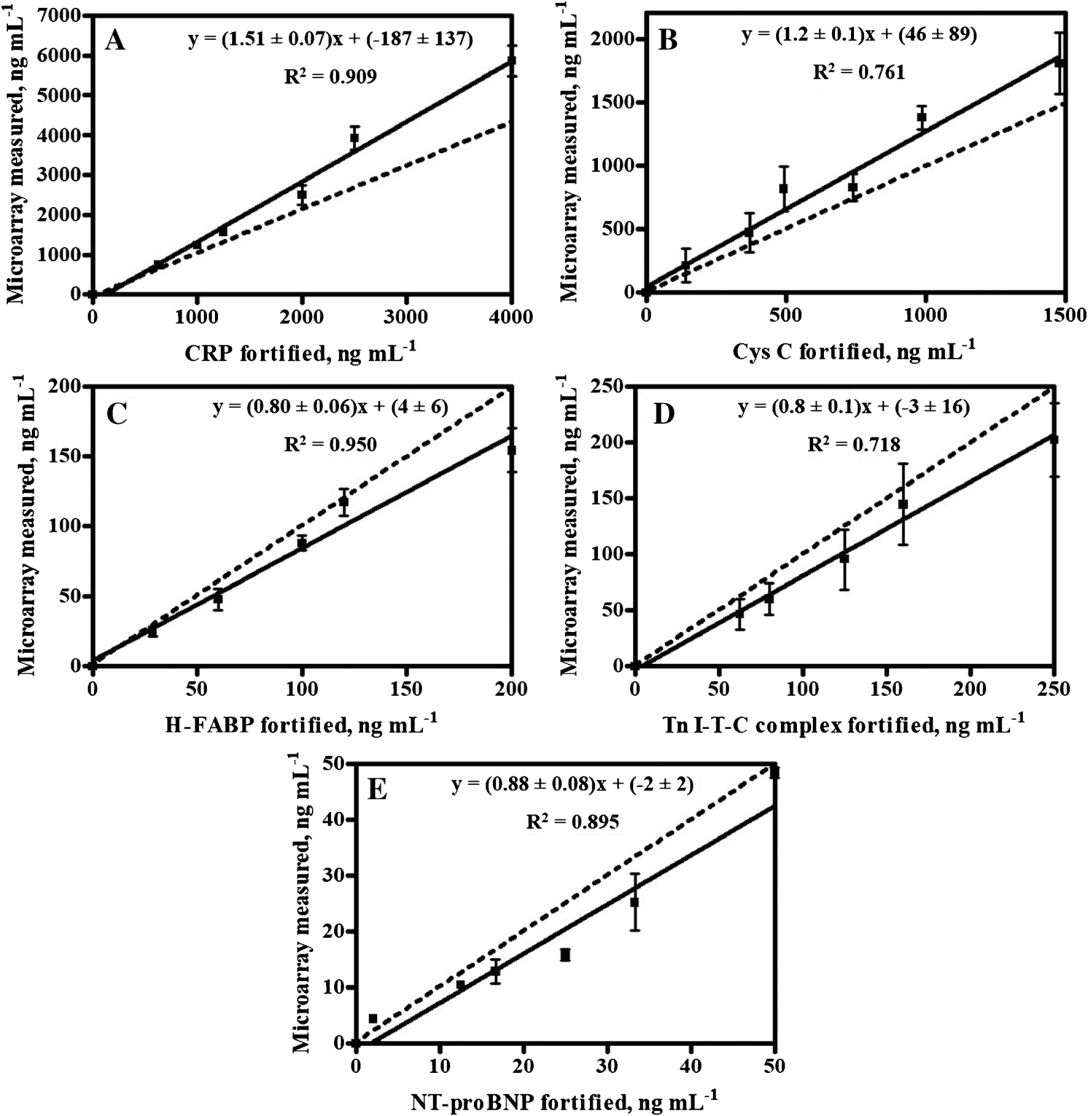
Correlation between the fortified and measured concentration values for **A** CRP, **B** Cys C, **C** H-FABP, **D** Tn I-T-C complex, and **E** NT-proBNP assays. CRP and Cys C are performed in one slide with 1/40 sample dilution, while H-FABP, troponin, and NT-proBNP are performed in another slide without sample dilution. The dotted line corresponds to a perfect correlation (slope = 1)

Thus, accuracy studies were assessed by measuring plasma samples fortified with all analytes at different concentrations. As it was mentioned, the samples were split in two parts, one of them diluted 1/40 for CRP and Cys C, and the other measured undiluted for the rest of the biomarker targets. The results shown in Fig. 4 correspond to the correlation found between the measured and the fortified concentration values. Despite the complexity of the sample and the multiplexed character of the technology, the accuracy was quite good. The recorded concentration values of CRP and CysC microarray tended to overestimate (slopes 1.5 and 1.2, respectively), while for NT-proBNP, H-FABP, and cTnI there was a slight underestimation (slopes between 0.8 and 0.9). With these results, we proceed to perform a small pilot study to get knowledge of the performance of the multiplexed chip while analyzing clinical samples.

### Clinical sample analysis

**Fig. 5:**
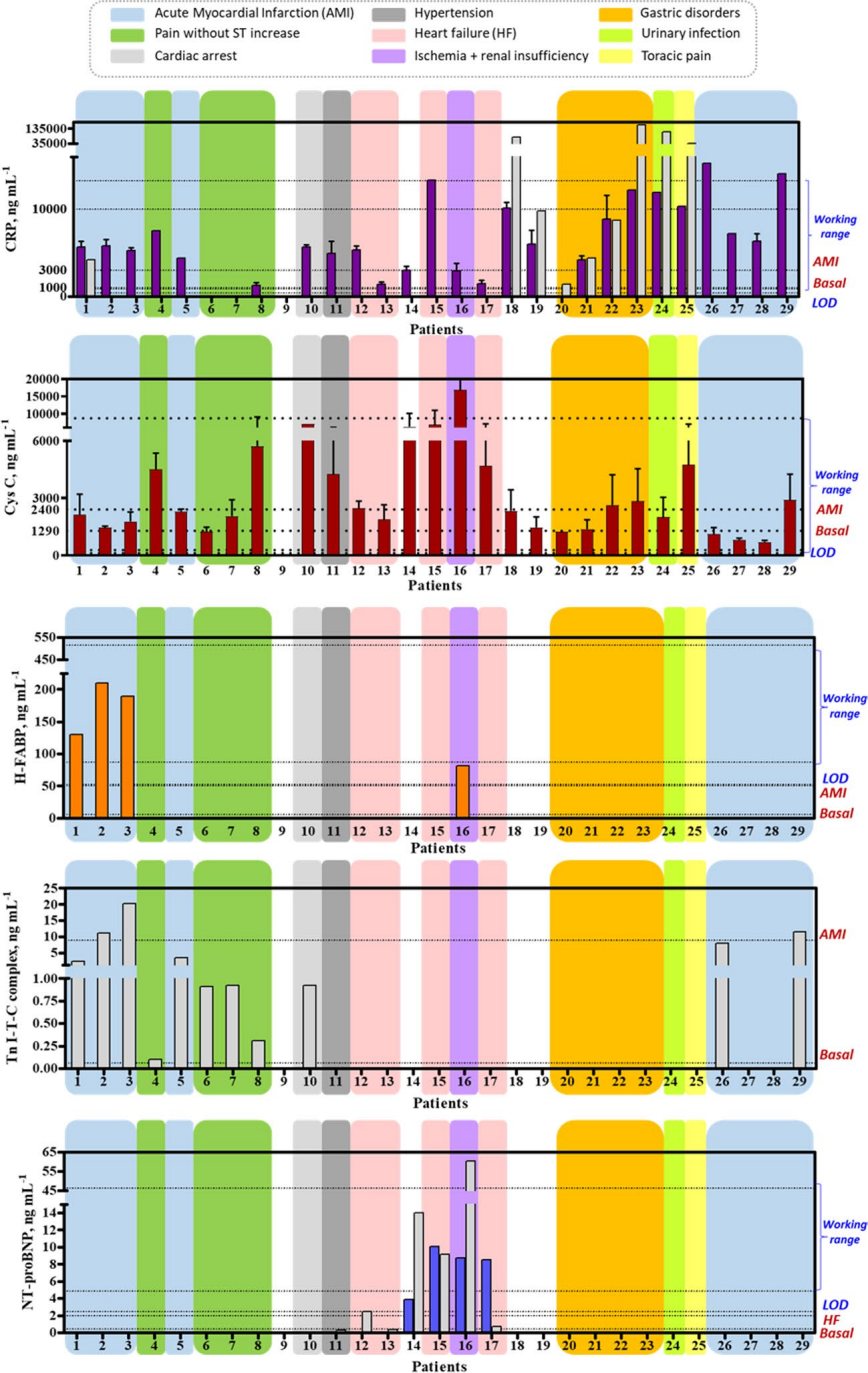
Results from the clinical plasma samples performed in the multiplexed microarray platform. Light gray bars correspond to measurements taken by the already characterized samples by IGTP. H-FABP, cTnI, and NT-proBNP were measured undiluted and CRP and Cys C was measured dilution 1/40 in PBST 0.15% casein pH 7.5. In the right y-axis, it is described the limit of detection (LOD) and working range, the basal concentration, and the established concentration found in the cardiovascular diseases for each biomarker

Clinical plasma samples obtained from patients showing different pathologies, with and without symptoms related to CVDs, were provided for the preliminary evaluation of the multiplexed microarray chip (see Table S1). The samples were partially characterized for some biomarkers using benchtop analyzers resulting from the clinical routine practice. Figure 5 shows the results from the quantification of the biomarker selected using both the microarray and the benchtop analyzers.

Levels of CRP over the basal levels were found in 25 out of 29 patients, which is not surprising, considering that CRP is an inflammatory biomarker which appears to be elevated in many pathologies. In contrast, CRP could only be quantified in 8 samples by the standard clinical laboratory methodologies. Probably, for most of the patients, the samples were not even analyzed due to the insufficient sample throughput capabilities of most of the clinical laboratories. All samples found positive on the benchtop analyzer were also found positive in the microarray except for sample 21, which CRP levels were close to the basal values. Regarding clinical interpretation of these results, the CRP is a biomarker related to inflammatory processes which explains why it was found elevated in many patients even if they did not suffer CVDs. Patients with CRP concentrations lower than 1000 ng mL^−1^ are classified as low risk, moderate for levels in the 1000–3000 ng mL^−1^ range, and high risk for concentrations higher than 3000 ng mL^−1^ [40]. Those patients diagnosed of AMI did show high levels of CRP and two of them (patients 10 and 13) were in very high risk. Also, according the CRP levels, patients 1, 18, and 19 were in high risk of having cardiovascular disorders, which is in accordance with the information related to their previous story of having chest pain without ST increase, cardiac arrest, and hypertension. Patients 14 and 16 diagnosed of HF were in high and very high risk and patients 26 and 27 who were passing the obstetrician control were in the high risk of CVD zone as well.

CysC was not analyzed in any of the patients by the standard clinical laboratory procedures. However, our multiplexed chip did find that the levels of this biomarker were over the basal levels in 18 patients, while for 5 of them were just at the cut-off. Moreover, for at least 8 of them, the concentration values were significative higher than the cut-off levels set for the case of an AMI event. Patients 2 and 21 had CysC levels corresponding to the basal levels which pointed out again that both patients had low probability to present cardiac problems. On the contrary, those patients diagnosed from AMI (10, 11, and 12) had similar levels and this fact underlines the doubtfully cardiovascular specificity of this marker. Even though, those patients suffering from cardiac arrest, HF, and thoracic pain (18, 16, 17, and 20) presented high levels of CysC above 2400 ng mL^−1^ which is the value assigned for high risk of CVD. Finally, patient 5 is the one with the highest level of CysC and he has ischemia and renal insufficiency. Increased levels of CysC are associated of kidney function and renal disorders, apart from risk of death and cardiovascular events in elderly persons.

For the case of H-FABP, it was not assessed using benchtop analyzers while it was included in the microarray platform developed in this article. H-FABP levels found in last patient 5 were above the AMI ones. Moreover, the H-FABP levels found in last patient 5 were above the AMI ones which shows some kind of relation. Furthermore, patients suffering AMI (6, 7, and 8) were in the same situation. Even though, other patients with also the same diagnostic their levels were not detected. It means they could be below AMI levels but above the basal levels. AMI levels are those ones representing high risk of infarction.

NT-proBNP results matched quiet well those of obtained by the standard clinical laboratory methods for the 29 samples analyzed. There were only a couple of discrepancies on patients 14 and 17. As it can be observed in the graph (Fig. 5), levels were found elevated and with risk of HF in 4 of the patients, while the benchtop analyzers did determine them elevated also in 4 of them. However, while patient 14 appears to have the NT-proBNP levels elevated according to the benchtop analyzer, the microarray did not. On the other hand, the microarray quantified elevated NT-proBNP levels in the plasma sample of patient 17 while the benchtop analyzer did not. NT-proBNP levels were found elevated in patients suffering from HF and ischemia with renal insufficiency (patients 5, 16, and17). It should also be noticed that high values of troponin I were assigned to patients without an AMI diagnostic which means that also analyzers provide incoherent results.

Unfortunately, as expected the chip was not able to quantify cTnI considered the golden biomarker in the case of AMI (see Fig. 5). From all patients analyzed, the benchtop analyzers were able to determine cTnI over the basal levels in 11 out of 29 patients, only 3 of them over the cut-off value related to AMI, while our microarray chip was not able to detect cTnI in any of these samples, due to the already observed lack of detectability of the immunoreagents used in this study.

Overall, we could say that in fact the microarray was able to give much more information and had superior performance regarding sample throughput capabilities. Thus, all 29 samples were measured and screened for the five biomarker targets on a short time, while with the benchtop analyzers each sample was measured only for particular biomarkers. For example, no data was collected at all regarding H-FABP and CysC levels of these patients and CRP levels were found elevated in much more patients than those analyzed with the benchtop equipment. Another important aspect to be noticed is that all the samples that did test positive with the microarray were also positive with the benchtop analyzers and in most cases a similar profile of response could be observed. For instance, for CRP it exists an agreement between both techniques for the samples that contained the higher or lower levels of CRP, similarly for NT-proBNP (Fig. 5).

## Conclusions

The multiplex platform developed allows the measurement of samples from patients with different pathologies by dividing the sample into two parts and analyzing them simultaneously in two different microarrays. The microarray provided useful and reliable data for most biomarkers in only 1.5 h and was able to analyze 29 samples simultaneously for the five biomarkers. The results show that the efficiency of this microarray in terms of the ability to measure multiple biomarkers in a large number of samples in a short time and the coherence of the results is much higher than that of any of the analyzers currently used in clinical laboratories. Despite some limitations in some aspects, the microarray could serve as a semi-quantitative tool for accurate risk stratification of patients and appropriate treatment. The microarray could then be used to quantify high CRP level patient samples that were not analyzed in the clinical laboratory. In addition, the microarray results were consistent with those obtained with analyzers for cases where measurements had been performed, and the disease had been diagnosed. The results were satisfactory even in the case of NT-proBNP, which had not reached the detectability baseline even though it was very close. Unfortunately, it was not possible to measure cTnI levels with the microarray as it was expected according to previous studies done with the same immunoreagents. Therefore, we can consider this multiplexed microarray as a semi-quantitative method useful for improving the diagnosis of cardiovascular disease for patients who are at different stages of the disease.

### Supplementary Information

Below is the link to the electronic supplementary material.

Supplementary file1 (DOCX 128 KB)
