# Supplementary material for: A multiplexed immunochemical microarray for the determination of cardiovascular disease biomarkers
Source: Mikrochim Acta. 2023 Dec 27;191(1):53. doi: 10.1007/s00604-023-06119-w (PMC10752916; doi:10.1007/s00604-023-06119-w)
Supplement: Supplementary file 1 — Supplementary file1 (DOCX 128 KB) [file 604_2023_6119_MOESM1_ESM.docx]

**Supplementary Information**

**A multiplexed immunochemical microarray for the determination of cardiovascular disease biomarkers**

Gloria Colom^1,2^, Alejandro Hernandez-Albors^1,2^, Jaume Barallat^3^, Amparo Galan^3^, Antoni Bayes-Genis^3^, J.-Pablo Salvador^2,1#^and M.-Pilar Marco^1,2^

^1^Nanobiotechnology for diagnostics (Nb4D), Department of Chemical and Biomolecular Nanotechnology, Institute for Advanced Chemistry of Catalonia (IQAC) of the Spanish Council for Scientific Research (CSIC), Jordi Girona 18-26, 08034 Barcelona, Spain.

^2^CIBER de Bioingeniería, Biomateriales y Nanomedicina (CIBER-BBN).

^3^ iCOR, Institut del Cor del Germans Trias i Pujol

Corresponding Author

#To whom correspondence should be addressed. Phone: 34 934006100. Fax: 34 932045904.

E-mail: [jpablo.salvador@iqac.csic.es](mailto:jpablo.salvador@iqac.csic.es)

**Buffers**

Unless otherwise indicated, phosphate buffer saline (PBS) is 0.01 M phosphate buffer in a 0.8% saline solution, pH 7.5. PBST consists of PBS with 0.05% Tween 20 with a pH of 7.5. Borate buffer is 0.25 M boric acid/sodium borate, pH 8.7, printing buffer is 150 mM sodium phosphate pH 8.5 with 0.01% sodium dodecyl sulphate and blocking buffer is PBS with 0.15% casein dissolved in 0.1 M NaOH and 0.05% Tween 20, pH 7.5.


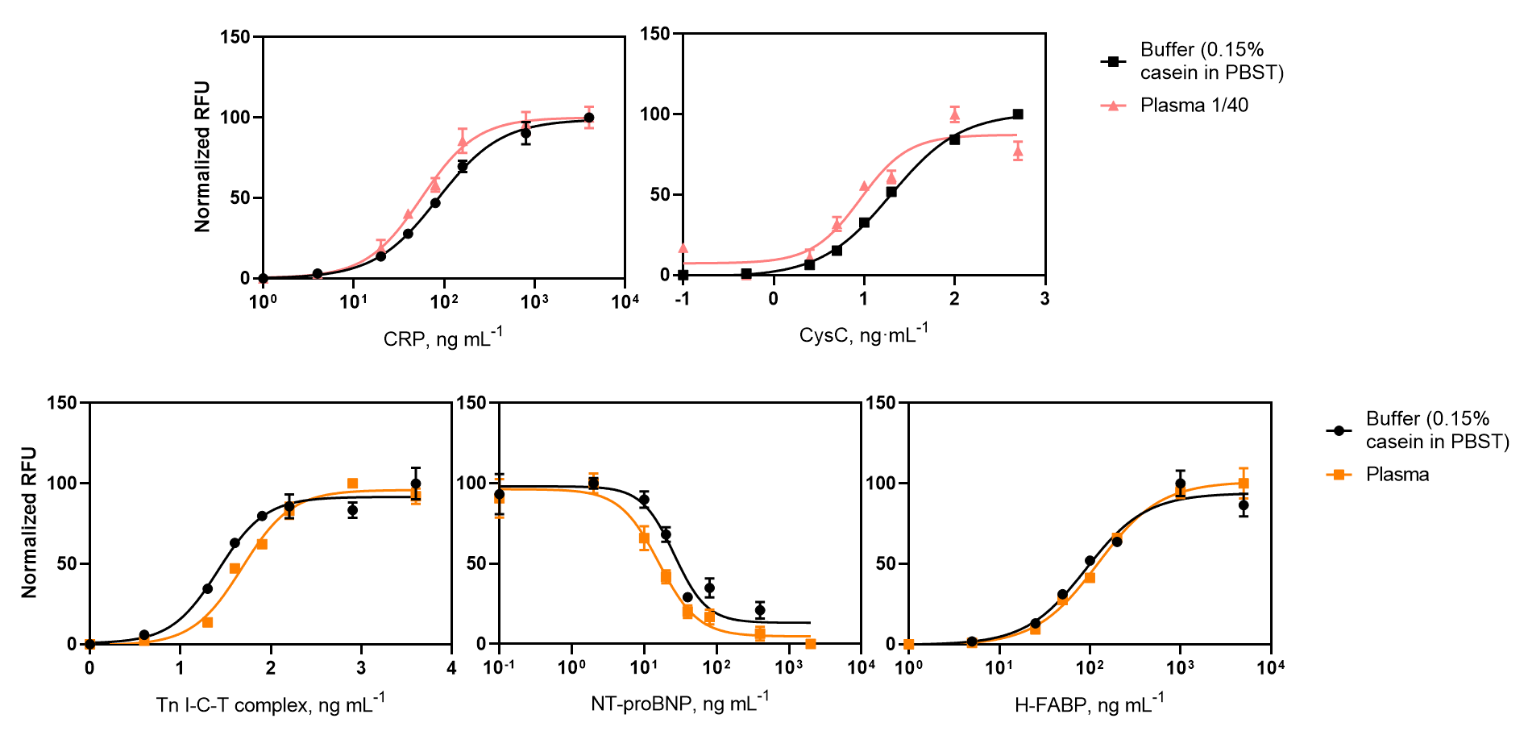


Figure S1. Matrix effect from plasma samples in the multiplexed microarray for each of the target analytes (CRP, CysC, Tn I-C-T, NT-proBNP and H-HFABP). For CRP and Cys, the matrix effect was evaluated diluting 1/40 plasma in buffer), Tn I-C-T, NT- proBNP and H-FABP was measured directly in plasma.

Table S1. Plasma samples from clinical patients were measured with the develop multiplexed microarray. CRP and CysC were measured a 1/40 dilution factor and cTnITC, NT- proBNP and H-FABP were measured directly in plasma. All values are expressed in ng/mL

|  | **CRP** | | **CysC** | | **hFABP** | | | **cTnITC** | | **NT-proBNP** | | |
| --- | --- | --- | --- | --- | --- | --- | --- | --- | --- | --- | --- | --- |
|  | **Multiplexed**  **microarray** | **Clinical**  **analyzer** | **Multiplexed**  **microarray** | **Clinical**  **analyzer** | **Multiplexed**  **microarray** | **Clinical**  **analyzer** | **Multiplexed**  **microarray** | | **Clinical**  **analyzer** | **Multiplexed**  **microarray** | **Clinical**  **analyzer** |  |
| **1** | 5689 ± 1094 | 4200 | 2141 ± 1500 | n.d. | 130 | n.d. | <LOD | | 2.32 | <LOD | n.d. |  |
| **2** | 5773 ± 1336 | n.d. | 1456 ± 113 | n.d. | 210 | n.d. | <LOD | | 11.18 | <LOD | n.d. |  |
| **3** | 5272 ± 582 | n.d. | 1779 ± 702 | n.d. | 189 | n.d. | <LOD | | 20.3 | <LOD | n.d. |  |
| **4** | 7551 | n.d. | 4514 ± 1185 | n.d. | <LOD | n.d. | <LOD | | 0.1 | <LOD | n.d. |  |
| **5** | 4419 | n.d. | 2294 ± 185 | n.d. | <LOD | n.d. | <LOD | | 3.52 | <LOD | n.d. |  |
| **6** | <LOD | n.d. | 1238 ± 347 | n.d. | <LOD | n.d. | <LOD | | 0.91 | <LOD | n.d. |  |
| **7** | <LOD | n.d. | 2059 ± 1201 | n.d. | <LOD | n.d. | <LOD | | 0.92 | <LOD | n.d. |  |
| **8** | 1262 ± 591 | n.d. | 5718 ± 4720 | n.d. | <LOD | n.d. | <LOD | | 0.31 | <LOD | n.d. |  |
| **9** | <LOD | n.d. | <LOD | n.d. | <LOD | n.d. | <LOD | | n.d. | <LOD | n.d. |  |
| **10** | 5680 ± 390 | n.d. | 7041 | n.d. | <LOD | n.d. | <LOD | | 0.92 | <LOD | 0.3 |  |
| **11** | 4951 ± 1955 | n.d. | 4253 ± 2582 | n.d. | <LOD | n.d. | <LOD | | n.d. | <LOD | 0.3 |  |
| **12** | 5316 ± 797 | n.d. | 2475 ± 522 | n.d. | <LOD | n.d. | <LOD | | n.d. | <LOD | 2.5 |  |
| **13** | 1403 ± 403 | n.d. | 1882 ± 1080 | n.d. | <LOD | n.d. | <LOD | | n.d. | <LOD | 0.4 |  |
| **14** | 3020 ± 825 | n.d. | 6272 ± 5392 | n.d. | <LOD | n.d. | <LOD | | n.d. | 3.9 | 14.0 |  |
| **15** | 13311 | n.d. | 6842 ± 7174 | n.d. | <LOD | n.d. | <LOD | | n.d. | 10.1 | 9.2 |  |
| **16** | 2919 ± 1511 | n.d. | 16890 ± 8030 | n.d. | 81 | n.d. | <LOD | | n.d. | 8.7 | 60.2 |  |
| **17** | 1485 ± 631 | n.d. | 4684 ± 4146 | n.d. | <LOD | n.d. | <LOD | | n.d. | 8.5 | 0.7 |  |
| **18** | 10113 ± 925 | 79000 | 2326 ± 1921 | n.d. | <LOD | n.d. | <LOD | | n.d. | <LOD | n.d. |  |
| **19** | 6024 ± 2737 | 9800 | 1465 ± 955 | n.d. | <LOD | n.d. | <LOD | | n.d. | <LOD | n.d. |  |
| **20** | <LOD | 1400 | 1250 | n.d. | <LOD | n.d. | <LOD | | n.d. | <LOD | n.d. |  |
| **21** | 4219 ± 762 | 4400 | 1384 ± 833 | n.d. | <LOD | n.d. | <LOD | | n.d. | <LOD | n.d. |  |
| **22** | 8869 ± 3884 | 8700 | 2633 ± 2745 | n.d. | <LOD | n.d. | <LOD | | n.d. | <LOD | n.d. |  |
| **23** | 12174 | 160000 | 2841 ± 2937 | n.d. | <LOD | n.d. | <LOD | | n.d. | <LOD | n.d. |  |
| **24** | 11910 | 114400 | 2013 ± 1781 | n.d. | <LOD | n.d. | <LOD | | n.d. | <LOD | n.d. |  |
| **25** | 10337 | 35000 | 4740 ± 3905 | n.d. | <LOD | n.d. | <LOD | | n.d. | <LOD | n.d. |  |
| **26** | 15222 | n.d. | 1115 ± 599 | n.d. | <LOD | n.d. | <LOD | | 7.9 | <LOD | n.d. |  |
| **27** | 7193 | n.d. | 812 ± 155 | n.d. | <LOD | n.d. | <LOD | | n.d. | <LOD | n.d. |  |
| **28** | 6308 ± 1588 | n.d. | 695 ± 143 | n.d. | <LOD | n.d. | <LOD | | n.d. | <LOD | n.d. |  |
| **29** | 14066 | n.d. | 2898 ± 1917 | n.d. | <LOD | n.d. | <LOD | | 11.51 | <LOD | n.d. |  |

Acute Myocardial infarction; Pain without ST increase; Cardiac arrest; Hypertension; Heart failure; Ischemia + renal insufficiency; Toracic pain; Gastric disorders; Urinary infection; Obstretic control; Unknown

<LOD, below the limit of detection, n.d. non detected
